# Bone marrow derived cells in adult skeletal muscle tissue in humans

**DOI:** 10.1186/2044-5040-3-12

**Published:** 2013-05-16

**Authors:** Anna Strömberg, Monika Jansson, Helene Fischer, Eric Rullman, Hans Hägglund, Thomas Gustafsson

**Affiliations:** 1 Department of Laboratory Medicine, Division of Clinical Physiology, Karolinska Institutet, Karolinska University Hospital Huddinge, 141 86, Stockholm, Sweden; 2 Department of Medicine, Center for Hematology and Regenerative Medicine, Karolinska Institutet, Karolinska University Hospital Huddinge, 141 86, Stockholm, Sweden; 3 Department of Medicine, Hematology Center, Karolinska Institutet, Karolinska University Hospital Huddinge, 141 86, Stockholm, Sweden

**Keywords:** Fluorescent *in situ* hybridization (FISH), Needle biopsies, Satellite cell niche, Vasculogenesis

## Abstract

**Background:**

During the past decade, several animal studies have demonstrated that in addition to local cells, cells from the bone marrow (BM) possess the ability to contribute to regeneration of injured skeletal muscle tissue. In addition, in mice, regular physical activity has been displayed to be a sufficient stimulus for BM-derived cell contribution to the muscle, indicating that this is part of the ongoing physiological remodeling of skeletal muscle. However, whether BM-derived cells participate in human skeletal muscle remodeling is not known. To this end, we analyzed the incorporation of BM-derived cells in healthy human skeletal muscle in women transplanted with male BM.

**Methods:**

Skeletal muscle biopsies were obtained from the m. vastus lateralis of women transplanted with male donor hematopoietic stem cells 6 to 12 years earlier. Healthy women served as controls. Immunohistochemical staining for skeletal muscle fibers, satellite cells (SCs) or endothelial cells (ECs) combined with fluorescent *in situ* hybridization (FISH) of X and Y chromosomes was used to identify cells of BM origin within the biopsies. Three dimensional confocal imaging was performed to demonstrate colocalization of Y chromosome and DAPI within muscle fibers. To further investigate whether BM-derived cells incorporate into the SC niche, myoblasts were extracted from the biopsies from the transplanted women, cultured, and analyzed using XY FISH and immunocytochemistry.

**Results:**

Three dimensional confocal imaging indisputably demonstrated colocalization of Y chromosome and DAPI within muscle fibers. Some Y chromosomes were found within centrally located nuclei. No Y chromosomes were detected in CD56+ SCs in the tissue sections nor in the myoblasts cultured from the extracted SCs. Y chromosome+ ECs were found in all sections from the transplanted subjects. No Y chromosomes were found in the skeletal muscle biopsies obtained from healthy control women.

**Conclusions:**

We demonstrate that BM-derived cells contribute to skeletal muscle fibers and ECs. Our results support that BM contribution to skeletal muscle occurs via direct fusion to muscle fibers, and that the contributing cells derive from the hematopoietic lineage. Thus, the present findings encourage further studies of the importance of this process for the physiological adaptation occurring throughout life.

## Background

In recent years, multipotent cells have attracted considerable interest because of their potential capacity to repair and remodel peripheral tissue. Skeletal muscle is an organ system that is under constant adaptation to changing work demands due to day-to-day physical activity and inactivity. This includes effects on the number of mitochondria, storage of substrates, enzyme activity levels, and changes in the contractility apparatus [1]. In addition to changes in muscle fibers, extracellular structures and supporting cells surrounding the fibers adapt to altered work demands [2]. The mature skeletal muscle cell is postmitotic; thus, muscle regeneration depends on the recruitment and fusion of progenitor cells with preexisting mature skeletal muscle fibers. The satellite cell (SC), which was identified in 1961 [3], is the residential stem cell of skeletal muscle tissue. However, the importance of this cell in the remodeling process was seriously recognized only recently [4,5]. It has been proposed that the SC pool is supplemented with progenitor cells from other sources, such as the bone marrow (BM) [6-8]. An alternative route suggested for BM-derived cell contribution to skeletal muscle is via direct fusion to muscle fibers in response to a physiological stimulus such as injury [9,10].

Another auxiliary structure with critical importance for skeletal muscle function is the capillary network surrounding each fiber, and alteration of the number of capillaries is a well-characterized extracellular remodeling process that occurs during skeletal muscle adaptation, both in humans and animals [1,11-13]. The earlier predominant view was that any changes in the number of vessels in adults were related to the proliferation of existing vessels, *i.e.*, angiogenesis or arteriogenesis. Currently, it has been shown that BM-derived endothelial progenitor cells (EPCs) contribute to vascular growth (vasculogenesis) in numerous animal models [14-16] and that the circulating level of these cells changes in human conditions of enhanced vascular formation [17-19]. Furthermore, several factors that stimulate vascular growth in skeletal muscle (*e.g.*, vascular endothelial growth factor-A, the angiopoietins, and erythropoietin) have been shown to stimulate both the angiogenic and vasculogenic processes [20-23]. Nevertheless, there are no data on the contribution of BM-derived cells to physiological vascular remodeling of skeletal muscle tissue in humans; however, data demonstrating that BM-derived cells differentiate into endometrial endothelial cells (ECs) in a human female after BM transplantation [24] support such hypothesis.

The aim of the current study was to investigate whether cells from the BM incorporate into skeletal muscle tissue in adult humans. This was achieved by analyzing skeletal muscle biopsies from women transplanted with male BM. Our hypothesis was that BM-derived cells contribute to the SC pool, skeletal muscle fibers and ECs in skeletal muscle tissue in humans.

## Methods

### Study subjects and experimental model

Three non-smoking, moderately active female subjects transplanted with male haematopoietic stem cells 6 to 12 years previously participated in the study. Their age, height and weight ranged between 28 to 36 yrs, 161 to 178 cm, and 69 to 83 kg, respectively. All patients were on post-transplant hormonal replacement therapy and in remission. Patient A had suffered from myelodysplastic syndrome, and was transplanted with BM from an unrelated donor. She had two children, a son and a daughter aged 11 and 13, respectively, at the time of obtaining the biopsy. Patient B had acute myeloid leukaemia, and had received BM-derived peripheral blood stem cells from her brother. Patient C had chronic lymphatic leukaemia, and was transplanted with BM from her brother. Neither patient B nor C had biological children. Two healthy, non-smoking, moderately active women served as controls. Their age, height and weight ranged between 35 to 36 yrs, 164 to 170 cm, and 58 to 65 kg, respectively. One of the control subjects had two sons aged 4 and 7, respectively, at the time of obtaining the biopsy. The other control subject had two children, a son and a daughter, aged 3 and 5. Prior to the study, the experimental protocol was explained to all subjects and informed written consent for participation was obtained. The study was approved by the Ethics Committee at Karolinska Institutet and conformed to the Declaration of Helsinki.

Muscle biopsy samples were obtained using the percutaneous needle biopsy technique from the vastus lateralis muscle at rest. The biopsies were immediately divided in two pieces; one piece was frozen within 10 to 15 s in isopentane cooled to freezing by liquid nitrogen and stored at −80°C until analysis, and the other was stored overnight in PBS/1% penicillin streptomycin at 4°C. For the control subjects the whole biopsy was frozen and stored at −80°C until analysis.

### Satellite cell isolation

About 30 mg of muscle tissue was put in a tube containing sterile PBS with 1% penicillin-streptomycin (Invitrogen AB, Stockholm, Sweden) and stored overnight at 4°C. After ~20 h, the biopsy was washed twice with PBS, cut in small pieces and digested in 5 mL 0.25% trypsin EDTA in 37°C, 5% CO_2_ with gentle agitation for 20 min. Undigested tissue was allowed to settle for 5 min and the supernatant was collected in DMEM-F12, with 20% FCS and 1% penicillin streptomycin. The cells were cultured until reaching ~70% confluence when a fraction of the cells was obtained for cytocentrifugation.

### Cytocentrifugation

The cells were suspended at a concentration of 10^5^ cells/mL and 100 μL were spun onto each glass slide (Superfrost/Plus microscope slides, Fisher Scientific, Pittsburgh, PA, USA). The slides were fixed in −20°C acetone for 10 min and then washed in PBS. Slides were blocked in PBS/4% BSA for 30 min in a humid chamber. A mouse anti-human desmin antibody (M0760, clone D33, Dako, Glostrup, Denmark) was added at a concentration of 1:200 in 1% BSA/PBS and the slides were incubated overnight in the cold room. The slides were washed in PBS before staining with the secondary rabbit-anti mouse Alexa488 antibody (A11059, Molecular Probes, Eugene, OR, USA) at 1:1000 in 1% BSA/PBS for 60 min in the humid chamber. After washing in PBS, the slides were mounted in Vectashield DAPI (406-diamidine-2-phenylidole-dihydrochloride)/antifade (Vector Laboratories Inc. Burlingame, CA, USA) and then analyzed for percentage of desmin positive cells.

### Immunohistochemistry

The frozen muscle biopsies were cut into 4 μm sections and placed on Superfrost/Plus microscope slides (Fisher Scientific). For serial sections, one section was put on each glass slide in a series of slides. The sections were fixed for 10 min in −20°C acetone, and then dried before three 3 min washes in PBS. The slides were blocked in PBS/4% BSA for 30 min in a humid chamber, and were then incubated with the primary antibody overnight at 4°C in the humid chamber. The antibodies used were mouse anti-human CD31 at 1:500 (M0823, Dako), mouse anti-human CD56 (NCAM) at 1:50, (347740, Becton Dickinson, San Jose, CA, USA), sheep anti-human laminin at 1:10000 (PC128, The Binding Site, Birmingham, UK), mouse anti-human Caveolin-3 (A-3) at 1:500 (sc-5310, Santa Cruz Biotechnology Inc. Santa Cruz, CA, USA) and mouse anti-human CD68 at 1:500 (M0718, Dako). After washing the slides in PBS, they were incubated in the secondary rabbit-anti mouse Alexa488 antibody (A11059, Molecular Probes) at 1:500 and/or the donkey anti-goat TexasRed at 1:300 (ab6883, Abcam, Cambridge, UK) for 60 min in room temperature, washed again and mounted in VectaShield with or without DAPI (Vector Laboratories). The staining was evaluated before fluorescent *in situ* hybridization.

### Fluorescent *in situ* hybridization (FISH)

After antibody staining, the glass slides were put in 2× saline sodium-citrate (SSC) buffer, and the cover slips were allowed to fall off. The chromosome enumeration probes (CEP) for the X and Y chromosomes (Vysis CEP X (DXZ1) SpectrumGreen or SpectrumOrange Probe and CEP Y (DYZ1) SpectrumAqua or SpectrumGreen Probe), were mixed with hybridization buffer according to the manufacturer (Abbott-Vysis Inc Downers Grove, IL, USA); 1.5 μL of the probe solution were added to a round cover slip (Thermo Fischer Scientific, Menzel GmbH & Co KG, Braunschweig, Germany), and the glass slide was then put on top of the cover slip. The cover slip was sealed around the edges with rubber cement, to prevent the probe solution from drying out during hybridization. Glass slides and probe mixture were denatured together in a Vysis HYBrite (Abbot Diagnostics). The melt temperature was set to 73°C for 2 min and hybridization temperature 38°C for 20 hours. When the hybridization was complete, the rubber cement was removed from the cover slips, and the slides were immersed in 2×SSC until the cover slips fell off. The slides were then washed in 0.4×SSC/0.3% Igepal, 72°C for 2 min and then 2×SSC/0.1% Igepal at room temperature for 30 s, and subsequently allowed to air-dry in the dark before mounting in VectaShield antifade (Vector Laboratories) with DAPI or propidium iodide as nuclear staining.

### Evaluation of immunohistochemistry and FISH staining

Evaluation and cell identification was done using an Olympus fluorescence microscope BH60 with appropriate filter set equipped with a CCD camera and connected to a Cyto-Vision image system (Applied Imaging Corp., San Jose, CA, USA) in which the results also were documented. Contribution of Y+ nuclei to skeletal muscle fibers was analyzed in sections stained with caveolin-3, while sections double-stained with laminin-1 and CD56 were analyzed for contribution to the SC pool. A cell stained with the marker CD56 containing a nucleus lying inside laminin-1 positive lamina was identified as an SC. Three sections from each subject, at least 80 μm apart, were completely examined by two independent investigators using an oil immersion objective with magnification 100×1.3. Some of the skeletal muscle fibers containing Y chromosomes were also evaluated using confocal microscopy. For evaluation of Y+ nuclei within ECs, two sections from each subject stained with CD31 were examined. To exclude for the possibility of the Y chromosome belonging to a leukocyte travelling within the vessel, serial sections stained with CD68 were performed and examined. After evaluating the sections for Y chromosomes, the sections were photographed using a Leica DMLA microscope equipped with a Leica DFC 420 C digital camera (Leica Microsystems AB, Sweden). The amount of myofibers and ECs per section was then calculated using the software Leica Qwin V. For myofibers, the tissue sections were photographed using the 5× objective. For ECs, the 20× objective was used and a picture was taken in a random location of the section. The area of the field was measured and the number of ECs was counted using Leica Qwin V. The area of the whole section was then measured at 5× magnification, and the number of ECs for the whole section was then estimated based on the assumption of equal distribution of ECs across the sections. The counting of ECs was performed in two different fields by two independent persons. For evaluation of Y chromosomes in the cytocentrifuged cells, the preparations were completely examined using an oil immersion objective with magnification 100×1.3. For each slide (n = 10 per subject), 4 visual fields were analyzed using the 40× objective for expression of desmin, a myogenic marker.

### Confocal microscopy

Tissue sections were visualized with a Zeiss LSM710 confocal system using a Plan-Apochromat 63×/1.40 Oil objective. Z-stacks were acquired sequentially using a 405 diode laser and the 488 laser line of the Argon laser to excite DAPI and SpectrumGreen, respectively. To determine whether a Y chromosome belonged to a muscle fiber nucleus, Y chromosome staining and DAPI nuclear staining had to colocalize and the nucleus of interest had to be on the same focal plane as the muscle membrane staining. Z-stacks are presented as maximum intensity projections. Three-dimensional rendering, maximum intensity projections and deconvolution were done with the AutoQuant X3 software.

## Results

### Immunohistochemistry and XY-FISH

**Figure 1:**
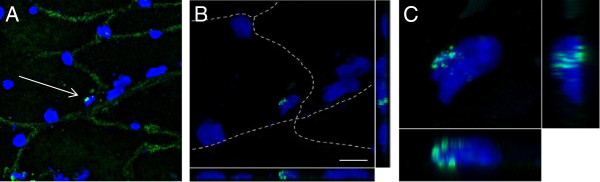
**Y chromosome-positive nuclei are incorporated into host muscle fibers.** Microphotographs show confocal images of combined FISH (Y chromosome, bright green, arrow) and indirect, fluorescent immunohistochemistry for caveolin-3 (green). Nuclear DNA was counterstained by DAPI. (**A**) Microphotograph of a muscle fiber section (width × height; 134.95 × 134.95 μm) depicting Y chromosome-negative host nuclei and a single Y chromosome-positive donor nucleus (arrow). Caveolin-3 staining is used to visualize muscle fiber membranes. (**B**) provides a Z-stack (width × height × depth; 67.48 × 67.48 × 5.148 μm; 13 optical sections) of the muscle fiber in (**A**). Dashed lines mark muscle fiber membranes indicating that the DAPI/Y chromosome nucleus is localized inside the muscle fiber. Size bar = 10 μm. (**C**) A high magnification Z-stack (15.83 × 15.83 × 6.007 μm; 15 optical sections) of the double-stained nucleus depicted in (**B**) clearly shows the integration of the Y chromosome DNA in the nucleus.

**Figure 2:**
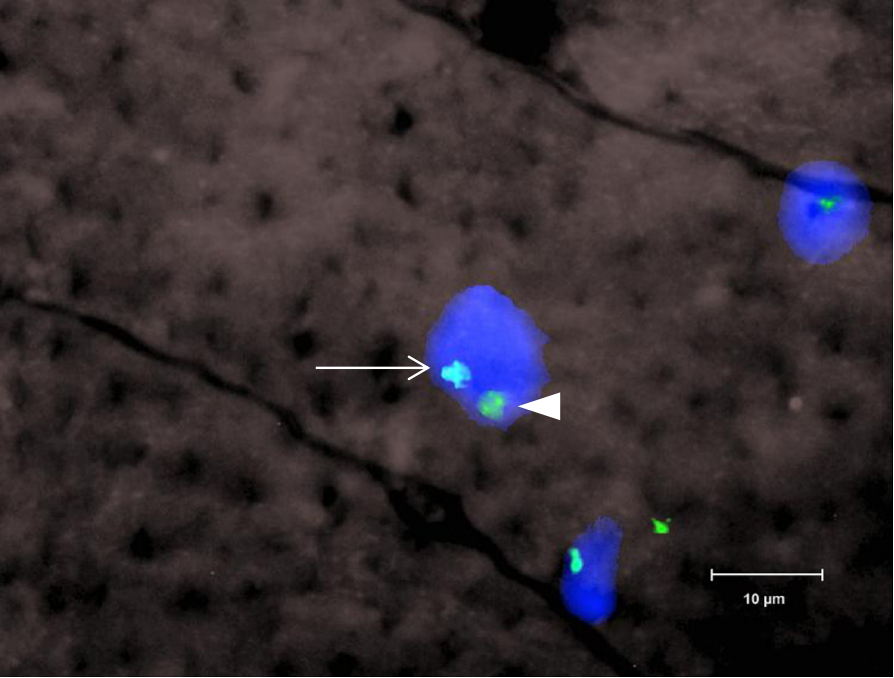
**Donor nucleus centrally located in host muscle fiber.** Y chromosome (turquoise, arrow) and X chromosome (green, arrowhead) inside host skeletal muscle fiber. Nuclei counterstained with DAPI. The section was examined by fluorescent microscopy with a 100× objective. Size bar = 10 μm.

**Figure 3:**
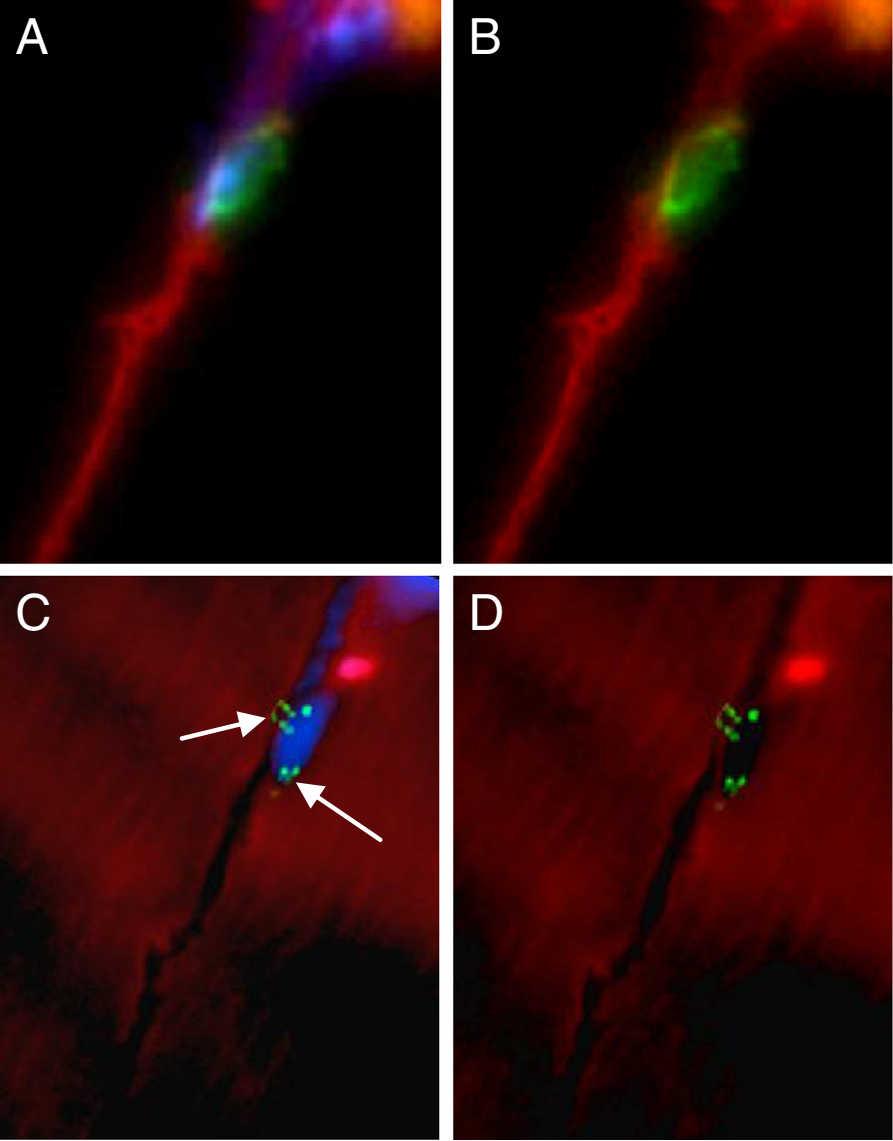
**Satellite cell containing two X chromosomes.** Immunofluorescent staining visualized by fluorescent microscopy shows XY-FISH combined with staining for the SC marker CD56. The section was examined with a 100× objective. (**A**,**B**) CD56 (green), together with laminin-1 (red), with and without DAPI. The CD56 positive satellite cell is located just beneath the basal lamina of the muscle fiber. (**C**,**D**) The same SC after XY-FISH, with and without DAPI, showing the presence of two X chromosomes (bright green, arrows).

**Figure 4:**
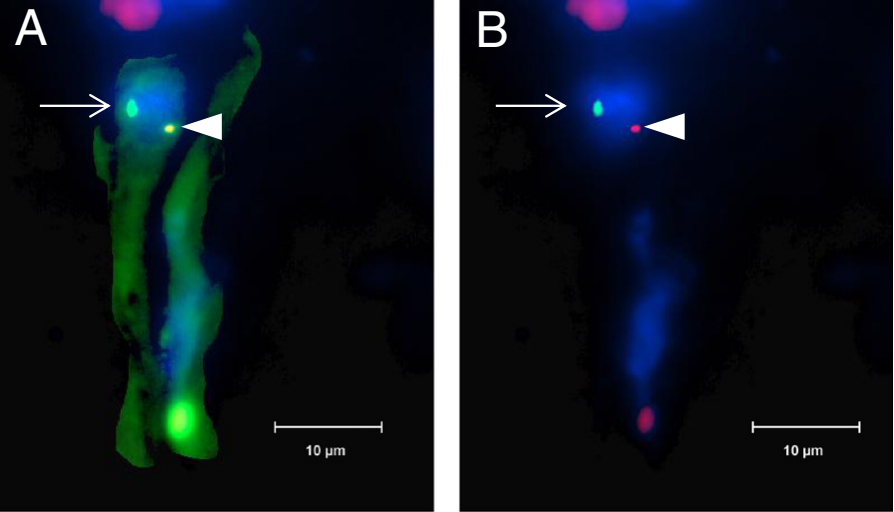
**Y chromosome-positive nuclei in endothelial cell.** Immunofluorescent staining visualized by fluorescent microscopy shows XY-FISH combined with staining for the endothelial marker CD31 (green). The section was examined with a 100× objective. (**A**) Y chromosome (bright green, arrow), together with X chromosome (red, arrowhead) inside endothelial cell stained for CD31 (green). Nuclear DNA was counterstained by DAPI. Size bar = 10 μm. (**B**) Same image without CD31-staining for better visualization of chromosome staining.

With caveolin-3 the sarcolemma was visualized, while laminin-1 stained the basal membranes. Three dimensional confocal imaging indisputably demonstrated colocalization of Y chromosome and DAPI within muscle fibers (Figure 1). Some Y chromosomes were found within centrally located nuclei (Figure 2). Laminin-1 together with CD56 staining showed SCs underneath the basal membrane of muscle fibers. No Y chromosomes were detected in CD56+ SC in the tissue sections analyzed (Figure 3). Y chromosome+ cells stained with CD31 were found in all sections from the transplanted subjects (Figure 4). In the sections from the skeletal muscle biopsies retrieved from the healthy controls, no Y chromosomes could be detected (no data shown).

### Number of Y chromosomes in skeletal muscle fibers and ECs

For subject A and B, 0.6% of the skeletal muscle fibers counted contained a Y chromosome within a nucleus. In subject C, 0.7% of the fibers had a Y chromosome inside a nucleus (Table 1). For ECs, some of the cells were cut so that the nucleus was not visible but the cells were positive for CD31. For subject A, 0.6% of the ECs contained a Y chromosome (0.9% per EC with visual DAPI staining). For subject B, 0.3% (0.5% of ECs with DAPI) of ECs had a Y chromosome. Subject C had 0.4% Y chromosome containing ECs (0.6% of ECs with DAPI) (Table 1).

**Table 1:** Detection of Y chromosomes in host skeletal muscle fiber and endothelial cell nuclei

	**No. of skeletal muscle fibers**	**No. of Y chromosomes in fibers**	**No. of ECs **	**No. of Y chromosomes in ECs**
**Subjects (n = 3)**	2796 (2287–3384)	18 (17–21)	3060 (2260–3982)	14 (9–24)

Number of skeletal muscle fibers and ECs counted per subject, together with the corresponding number of Y chromosomes. The total number of fibers, ECs and Y chromosomes is reported as an average (range) for the three subjects analyzed.

### Cytocentrifugation

**Figure 5:**
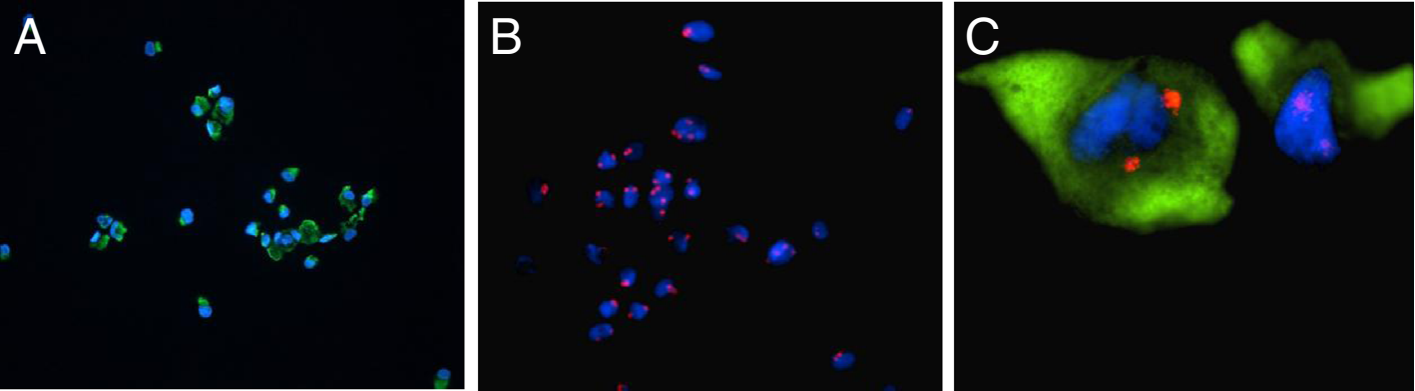
**Immunofluorescent and FISH analysis of cytocentrifuged myoblasts from satellite cell extraction.** (**A**) Desmin staining (green) before XY-FISH, 20× magnification. (**B**) Myoblasts after XY FISH. X chromosomes were stained red and Y chromosomes green. All cells were negative for the Y chromosome, 20× magnification. (**C**) Myoblasts stained with desmin (green) containing two X chromosomes (red), 100× magnification. The nuclei were counterstained with DAPI.

About 80% of the cytocentrifuged cells from the SC extraction were positive for desmin. These cells are myoblasts; no intact muscle fibers were obtained from the biopsies. None of the slides analyzed had a single cell containing Y chromosomes (Figure 5).

## Discussion

In the current study, we demonstrated that Y chromosomes are present within myofibers and in cells with an endothelial phenotype/morphology in women transplanted with male hematopoietic stem cells. No Y chromosome positive SCs were detected in the tissue sections or in the cytocentrifuged cell population derived from SC isolation.

In tissue sections stained using XY FISH together with either caveolin-3, which stained the sarcolemma, or laminin-1, which stained the basal membrane, epifluorescence microscopy showed Y chromosomes present within myofibers. To further demonstrate colocalization of Y chromosome and DAPI nuclear staining within muscle fibers, three dimensional confocal imaging was performed. Interestingly, some of the Y chromosomes found within myofibers were observed in centrally located nuclei, which is indicative of a regenerating fiber [25]. In the present study, no Y chromosomes were detected in the SC niche in tissue sections stained with laminin-1 together with CD56. Further, not a single Y chromosome was detected in desmin positive myoblasts in the cell cultures from the isolated SCs. In a study performed in mice, BM-derived cells were shown to be incorporated into the SC niche [8]. The current data does not support such a mechanism; instead it suggests that BM-derived cells fuse directly with the muscle fibers rather than passing through a myogenic stem cell stage. This is also coherent with a very well performed study in mice, were an estimated number of 375,000 SCs were assayed to determine the mechanism behind haematopoietic stem cell contribution to skeletal muscle [26]. In their experiments not a single SC was shown to derive from the BM, and BM contribution to skeletal muscle occurred through myeloid cell fusion to muscle fibers [26].

The observation of Y chromosomes in cells stained with CD31 support the notion that circulating cells incorporate into the skeletal muscle endothelium in humans. Importantly, to exclude the possibility of the Y chromosome belonging to a leukocyte travelling within the vessel only CD31+ Y chromosome+ cells without a CD68+ cell on the same localization in a serial section were counted. Since the discovery by Asahara et al. in 1997 of the formation of ECs from human BM mononuclear cells in mice, a large number of articles on EPCs have been published in various animal settings. This includes the contribution of EPCs to neovascularization of myocardium, brain and hindlimb in rodents after induced ischemia [16,27,28]. Still, even though the circulating level of EPCs is known to change in human conditions of enhanced vascular formation, to our knowledge only one earlier report exists of actual contribution to endothelium in humans, where BM-derived cells were shown to differentiate into endometrial ECs in a human female [24]. Our study provides novel data regarding the contribution of BM-derived cells to skeletal muscle tissue in humans.

In the present study we have not been able to elaborate on the functionality of these incorporated cells. Because of the low number of BM-derived cells found in our study, one may think that this is a process with no physiological relevance. However, based on earlier findings in mice [8,29], it is tempting to speculate on whether their number would have been higher if these women had been subjected to remodeling stimuli, such as habitual endurance-type exercise. Importantly, the number of skeletal muscle fibers containing Y chromosomes in our study is consistent with levels found in non-exercised mice [8,26], in which the number of cells shown to increase in response to increased muscle activity from muscle overload, forced downhill running [29], and voluntary exercise [8]. Moreover, the role of EPCs in vascular formation and repair is a largely unresolved question; do they act in a paracrine fashion to supply local cells with growth factors, or do they form ECs themselves? For instance, the beneficial effects of the injection of EPCs into patients with myocardial infarction are measured using markers of cardiac function, such as left ventricular ejection fraction, which does not reveal whether the injected cells have actually formed ECs [30,31].

One could claim that the XY cells found in these women derive from male offspring. However, only one of the women had biological children and she gave birth to her son 11 years before the beginning of this study. In addition, not a single Y-chromosome containing cell was found in sections from the control women that had given birth to boys. Furthermore, as the women who participated in our study had received either BM transplantation or peripheral blood stem cells, the exact phenotype/origin of the BM-derived stem cells incorporated into muscle fibers remains undetermined. Regarding the BM-transplanted women, donor-derived cells may be mesenchymal stem cells derived from the BM stroma or hematopoietic stem cells. The woman transplanted with peripheral blood stem cells, however, exhibited the same level of donor-derived cells in her muscle tissue, indicating that the ability to form myonuclei or ECs lies in the hematopoietic lineage. Nevertheless, the contribution of mesenchymal stem cells to skeletal muscle tissue in the BM-transplanted women cannot be excluded in this study. Another issue is whether irradiation administered prior to BM transplantation affects the tissues and the systemic environment in a manner that causes the incorporation of BM-derived cells into tissues. However, irradiation was shown not to be a prerequisite for BM-derived cell contribution to myofiber regeneration in a study performed in mice [29].

## Conclusions

In conclusion, the contribution of BM-derived cells to skeletal muscle fibers and ECs was demonstrated by obtaining skeletal muscle biopsies from women transplanted with male BM. The extent of contribution to muscle fibers was similar to the levels seen in transplanted mice not exposed to injury or exercise [8,26]. Our results support that BM contribution to skeletal muscle occurs via direct fusion to muscle fibers, and that the contributing cells derive from the hematopoietic lineage. The present study encourages further studies of the importance of this process for the physiological adaptation occurring throughout life.

## Consent

Written informed consent was obtained from the subjects for publication of this manuscript. A copy of the written consent is available for review by the Editor-in-Chief of this journal.

## Abbreviations

BM: Bone marrow; BSA: Bovine serum albumin; DAPI: 406-diamidine-2-phenylidole-dihydrochloride; EC: Endothelial cell; EPCs: Endothelial progenitor cells; FCS: Fetal calf serum; FISH: Fluorescent in situ hybridization; PBS: Phosphate buffered saline; SC: Satellite cell.

## Competing interests

The authors declare that they have no competing interests.

## Authors’ contributions

AS designed the study, collected and analyzed the material, interpreted the data and drafted the manuscript. MJ contributed to the study design, and participated in the acquisition and interpretation of data. HF analyzed and interpreted the data. ER obtained the muscle biopsies and helped to draft the manuscript. HH recruited the study subjects and contributed to the study design. TG conceived the study, and contributed to the design and coordination of the study and drafted the manuscript. All authors read and approved the final manuscript.
